# Autologous whole blood clot formation with actigraft in patients receiving oral anticoagulant therapy: an ex vivo feasibility study

**DOI:** 10.1007/s11239-026-03321-4

**Published:** 2026-05-24

**Authors:** Barry Frank Jacobson, Elise Schapkaitz, Melanie Mc Cree

**Affiliations:** 1 Department of Surgery, Faculty of Health Sciences, University of Witwatersrand Medical School, Johannesburg, South Africa; 2 Department of Molecular Medicine and Hematology, Faculty of Health Sciences, University of Witwatersrand Medical School, 7 York Rd, Parktown, Johannesburg Gauteng, South Africa; 3 National Health Laboratory Service, Johannesburg, South Africa

**Keywords:** Occlusive dressings, Wounds and injuries / therapy, Haemostatics, Anticoagulation

## Abstract

The clotting characteristics of autologous whole blood clots for chronic wound management in patients on anticoagulant therapy are not well defined. An exploratory ex vivo study was performed using the ActiGraft autologous whole blood clot (RedDress Ltd, Haifa, Israel) in patients receiving oral anticoagulant therapy. Twenty-five participants were eligible for inclusion and stratified into three age-matched cohorts: vitamin K antagonist-warfarin (*n* = 9), factor Xa inhibitor- rivaroxaban (*n* = 6), and controls not receiving anticoagulation (*n* = 10). The primary endpoint was clot formation time. Clot formation time was defined as the time point at which no fluid movement was observed. In the study population (median [interquartile range] age 47 [42–52] years), the mean clot formation time was 10.5 ± 2.6 min (reference:8–12 min). Mean clot formation time was significantly different across groups (*p* < 0.001). Compared with controls (8.4 ± 1.2 min), clot formation time was significantly prolonged in both the warfarin group (11.3 ± 2.3 min; *p* < 0.002) and the rivaroxaban group (12.7 ± 2.4 min; *p* < 0.001), with no significant difference between anticoagulant groups (*p* = 0.297). Nonetheless, complete, stable clots were consistently formed within the expected time frame in all samples across all groups. This illustrates the feasibility of ActiGraft autologous whole blood clot in patients receiving therapeutic anticoagulation. Further studies are indicated in patients with chronic wounds to assess broader safety and efficacy.

**Graphical Abstract:**

**Figure:**
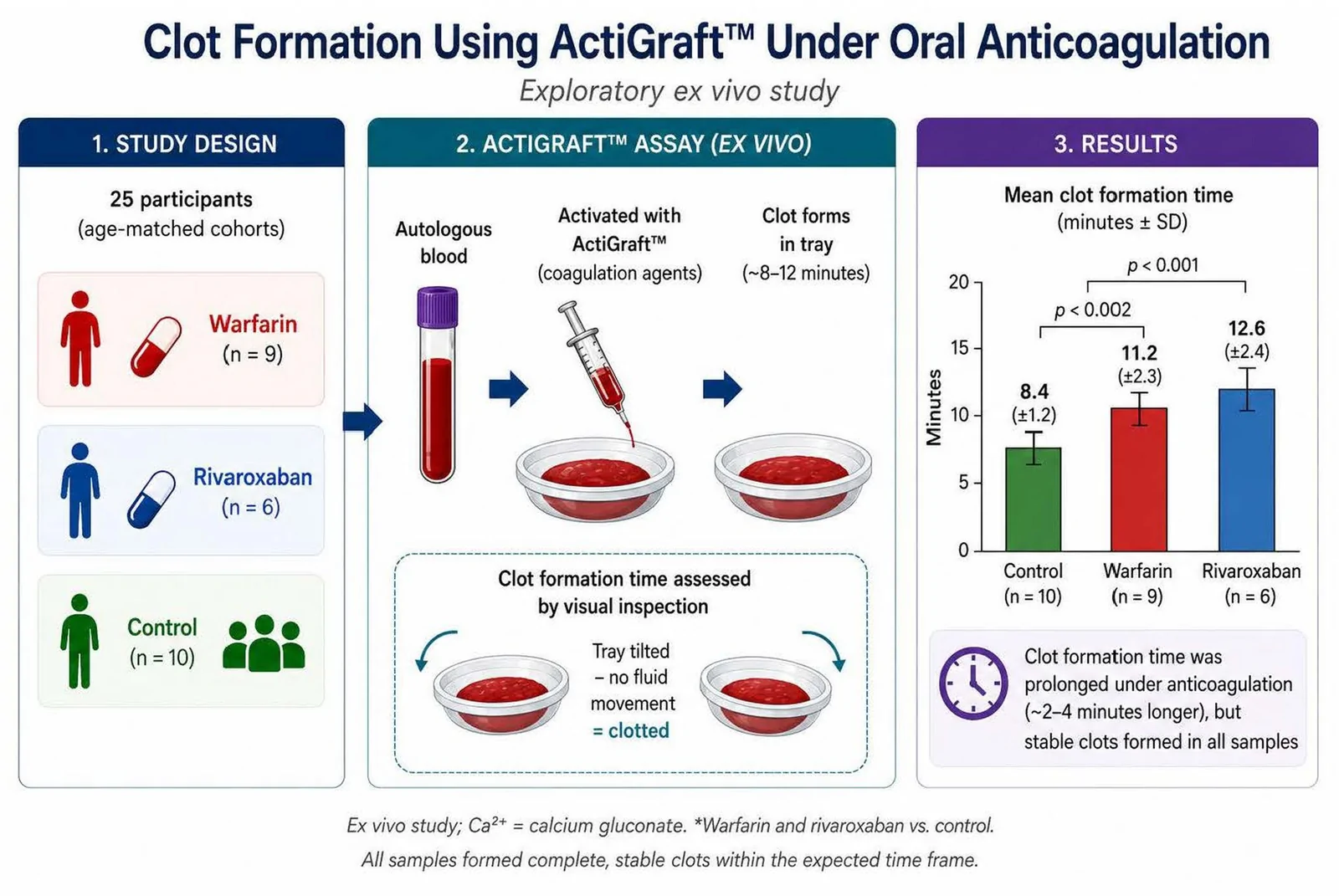

**Supplementary Information:**

The online version contains supplementary material available at https://doi.org/10.1007/s11239-026-03321-4.

## Introduction

Chronic wounds, defined as wounds that fail to progress through the normal healing process and remain open for more than one month, affect over 2% of the worldwide population [1–3] with prevalence increasing with age [4, 5]. These wounds frequently resist conventional treatment modalities, resulting in prolonged morbidity and substantial clinical and economic burden. In recent years, ActiGraft (RedDress Ltd, Haifa, Israel), an autologous whole blood clot, has emerged as a promising adjunctive therapy, leveraging the patient’s own blood to create a bioactive dressing that mimics the extracellular matrix (ECM) and provides a provisional scaffold that promotes wound healing [6–8].

ActiGraft has demonstrated efficacy in the treatment of chronic wounds of various etiologies [9–11]. This technology utilizes powdered coagulation agents to accelerate the clotting cascade, promoting rapid formation of a whole blood clot that is subsequently applied as a topical wound dressing. The therapeutic activity of ActiGraft is rooted in the complex biological mechanisms of coagulation, which govern fibrin clot formation and play a central role in the initiation and regulation of wound healing.

Clot formation is a critical determinant of ActiGraft structure and function, directly influencing the clot’s bioactivity, mechanical integrity, and adherence to the wound bed. However, patients receiving oral anticoagulation therapy present a potential challenge to ActiGraft preparation. Anticoagulants, including vitamin K antagonists and direct oral anticoagulants (DOACs), are widely prescribed for conditions such as atrial fibrillation, venous thromboembolism, and mechanical heart valve disease [12]. By altering coagulation parameters, these agents may affect clot formation and, consequently, the performance of ActiGraft as a wound-healing modality.

Physiological coagulation is mediated by a tightly regulated cascade of enzymatic reactions that culminate in fibrin clot formation, stabilizing the initial platelet plug at sites of vascular injury [13]. This cascade comprises intrinsic, extrinsic, and common pathways, ultimately leading to the conversion of fibrinogen to fibrin by thrombin, forming a stable clot [14]. Anticoagulant therapies interfere with this process at distinct points. Vitamin K antagonists, such as warfarin, inhibit the synthesis of vitamin K-dependent clotting factors II, VII, IX, and X, thereby prolonging coagulation times and reducing thrombin generation [15]. Direct oral anticoagulants, including factor Xa inhibitors such as rivaroxaban, selectively inhibit factor Xa activity, preventing thrombin generation and subsequent fibrin formation [16]. These pharmacologic effects may delay clot initiation and alter clot structure or stability, which are essential for the effective use of ActiGraft as a bioactive wound dressing.

As the use of anticoagulant therapy becomes increasingly common, particularly among older individuals with cardiovascular comorbidities, it is increasingly important to understand how these agents influence clot formation during ActiGraft preparation. To date, no prospective clinical trial of ActiGraft therapy has been specifically designed to evaluate efficacy or safety in patients receiving therapeutic anticoagulation, and available evidence is derived from mixed patient cohorts without stratified analysis by anticoagulant use. Consequently, the feasibility of reliable ActiGraft clot formation on oral anticoagulation remains insufficiently characterized.

Indirect support for the feasibility of autologous clot-based biomaterials under anticoagulation comes from platelet-rich fibrin studies in dental surgery, which report that fibrin-based matrices can be generated and used safely in patients receiving vitamin K antagonists or DOACs [17]. These findings, however, cannot be directly extrapolated to whole blood clot dressings or chronic wound settings.

In this study, we aimed to evaluate blood clotting characteristics in patients receiving anticoagulant therapy using a bedside ActiGraft system. We assessed whole blood clot formation across anticoagulant classes to determine the feasibility of ActiGraft preparation in this population. By examining how different anticoagulant therapies influence clot dynamics in an ex vivo setting, this study seeks to inform ActiGraft preparation protocols and patient selection.

## Materials and methods

### Study design

This was an ex vivo observational exploratory study designed to evaluate the effect of oral anticoagulant therapy on whole blood clot formation using the ActiGraft system. The study was structured as a three-cohort, age-matched comparative analysis and included 30 participants stratified into three groups in a 1:1:1 ratio: (1) patients receiving a vitamin K antagonist (Warfmadin, Sanofi^®^, Turkey), (2) patients receiving a factor Xa inhibitor (rivaroxaban, IXarola, Sanofi^®^, South Africa), and (3) control patients not receiving anticoagulant therapy. Participants were enrolled during routine clinical visits between 1 April and 30 June 2025 after providing written informed consent. Participants were enrolled at the Anticoagulation clinic in the Department of Haematology at Charlotte Maxeke Johannesburg Academic Hospital in Gauteng, South Africa, and all ex vivo clot formation assays were performed on site using the ActiGraft system.

### Participant selection

Eligible participants were adults aged 18 years or older who were either receiving anticoagulant therapy with warfarin (vitamin K antagonist) or rivaroxaban 20 mg (factor Xa inhibitor) for at least seven days or were not receiving any anticoagulant therapy. Age matched controls were selected from the hospital staff during the study period. These individuals were not receiving anticoagulant therapy and had no known coagulopathy. Age matching was performed to reduce the potential confounding effect of age on coagulation parameters; however, no additional matching for comorbidities was undertaken given the exploratory nature of the study. Participants were not required to have an active acute or chronic wound, and blood samples were collected independently of wound status.

Exclusion criteria included inability to provide the required blood volume of 36 mL, known bleeding disorders, treatment with intravenous heparin or direct thrombin inhibitors (to ensure a homogenous study population), subtherapeutic anticoagulation levels, and inability to read or understand the informed consent form.

### Sample collection and processing

For each participant, 36 mL of peripheral blood was collected into acid citrate dextrose solution A (ACDA) vacuum tubes (Becton-Dickinson, Oxford, UK). In participants receiving anticoagulant therapy, an additional 3.2% sodium citrate (Becton-Dickinson, Oxford, UK) blood sample was collected for laboratory assessment of anticoagulation status, including measurement of the international normalized ratio (INR) for those receiving vitamin K antagonists or anti-factor Xa levels four hours after the morning dose for those receiving factor Xa inhibitors. The samples were processed at the Lancet laboratory using a Siemens Atellica COAG 360 (Siemens Healthcare Diagnostics, Tarrytown, NY, USA) analyser. Laboratory values were collected to document therapeutic anticoagulant exposure.

Blood samples were processed ex vivo at room temperature using the ActiGraft system to generate two whole blood clots per participant. Clot formation was assessed by visual inspection at seven predefined time points following activation: 5, 7, 8, 9, 10, 11, and 12 min. At each time point, the clotting tray was gently tilted to assess fluid movement. Clot formation time was defined as the time point at which no fluid movement was observed, indicating completion of clot formation (reference 8–12 min). For each participant, clotting time was calculated as the mean of the two technical replicates and used for all subsequent analyses.

### Statistics

Clinical and laboratory data were analyzed using Statistica 13.2 software (Palo Alto, California, USA). Categorical variables were summarized using frequencies and percentages. Continuous variables were summarized using means and standard deviations (SD) for normally distributed data or medians and interquartile ranges (IQR) for non-normally distributed data. Agreement between technical replicates was assessed using a paired t-test. Comparisons of clot formation time across the three groups were performed using a parametric one-way ANOVA test with Scheffe’s post hoc test. Comparisons between the warfarin and rivaroxaban groups were performed using an unpaired t-test. Pooled analyses of anticoagulated groups versus controls were performed as a secondary comparison using an unpaired t-test. Comparisons of non-normally distributed continuous variables, including age, were performed using the Kruskal–Wallis test.

Categorical variables were compared using Fisher’s exact test. All statistical analyses were two-sided and conducted using a significance level (alpha) of 0.05. Given the exploratory nature of the study, no formal sample size calculation was performed.

## Results

### Participant characteristics

Thirty participants were enrolled, with 10 individuals assigned to each group. Of these, 25 met eligibility criteria and were included in the final analysis. Exclusions comprised one participant with subtherapeutic warfarin levels, two participants with subtherapeutic rivaroxaban levels, and two rivaroxaban-treated participants without available anti–factor Xa measurements. The median age of the analyzed cohort was 47 years (interquartile range 42–52) and did not differ significantly between groups. Most participants were of African ethnicity (23/25, 92%), reflecting the hospital population; the distribution of ethnicities was comparable across groups. Baseline demographic and laboratory anticoagulation characteristics are presented in Table 1.

**Table 1:** Patient demographics

Characteristic	Vitamin K antagonist (Warfarin)	Factor Xa inhibitor (Rivaroxaban)	Control	*P* value
	*n* = 9	*n* = 6	*n* = 10	
Age, year, median [IQR]	50 [44–59]	52 [42–63]	44 [40–50]	0.298
*Sex*,* n (%)*				
Female	5 (56%)	2 (33%)	7 (70%)	0.404
Male	4 (44%)	4 (67%)	3 (30%)	
*Race*,* n (%)*				
Black African	9 (100%)	4 (67%)	10 (100%)	0.050
Other^1^	0 (0%)	2 (33%)	0 (0%)	
INR, median [IQR]^2^	2.8 [2.0-3.2]	NA	NA	NA
Anti Xa, (ng/mL) median [IQR]^3^	NA	201.5 [164.8-224.8]	NA	NA

^1^Other included 1 White and 1 Coloured.

^2^For reference, therapeutic INR ranges are typically 2.0–3.0 for venous thromboembolism and atrial fibrillation and 2.5–3.5 for patients with mechanical valve replacement.

^3^Expected peak anti–factor Xa levels for rivaroxaban are dose dependent and reported as 189–419 ng/mL for 20 mg.

### Clot formation time

Clot formation times did not differ between the two technical replicates (*p* = 0.262); therefore, the mean value per participant was used for all subsequent analyses (Table 2). The overall mean clot formation time was 10.5 ± 2.6 min. Clot formation time differed significantly across groups (*p* < 0.001). All ex vivo clotting assays yielded evaluable results, and complete clot formation was achieved in all samples within the predefined observation window. Mean clotting time was longer in the oral anticoagulant group compared with controls (11.9 ± 2.3 min vs. 8.4 ± 1.2 min, *p* < 0.001). Similarly, clot formation time was significantly prolonged in both the warfarin group (11.3 ± 2.3 min; *p* < 0.002) and the rivaroxaban group (12.7 ± 2.4 min; *p* < 0.001), compared with controls (8.4 ± 1.2 min) (Table 3). Despite this statistically significant prolongation, consistent whole blood clot formation was achieved across all anticoagulated samples, indicating preserved feasibility of clot generation under oral anticoagulant therapy. No significant difference in clotting time was observed between the warfarin and rivaroxaban groups (*p* = 0.297).

**Table 2:** Individual and average time to clot formation

Characteristic	Vitamin K antagonist (Warfarin)	Factor Xa inhibitor (Rivaroxaban)	Control	*P* value
	*n* = 9	*n* = 6	*n* = 10	
Clotting time 1 mean ± SD (minutes)	11.1 ± 2.4	13.3 ± 3.3	8.0 ± 1.2	0.001
Clotting time 2 mean ± SD (minutes)	11.6 ± 2.5	12.0 ± 2.0	8.7 ± 1.3	0.004
Average Clotting time mean ± SD (minutes)	11.3 ± 2.3	12.7 ± 2.4	8.4 ± 1.2	0.001

**Table 3:** Average time to clot formation among anticoagulant groups

	Vitamin K antagonist (Warfarin)	Factor Xa inhibitor (Rivaroxaban)	*P* value
	*n* = 9	*n* = 6	
Average Clotting time mean ± SD (minutes)	11.3 ± 2.3	12.7 ± 2.4	0.297

	Anticoagulant group	Control	*P* value
	*n* = 15	*n* = 10	
Average Clotting time mean ± SD (minutes)	11.9 ± 2.3	8.4 ± 1.2	0.001

## Discussion

Patients with chronic persistent wounds frequently present with comorbidities that complicate wound management, including cardiovascular disease and venous thromboembolism. Some of those conditions are commonly treated with long-term oral anticoagulant therapy. Because ActiGraft relies on the formation of a blood clot to function as a bioactive wound dressing, there is a clear biological rationale to question whether anticoagulation could impair clot generation and limit therapeutic feasibility. This concern is particularly relevant given the widespread use of vitamin K antagonists and direct oral anticoagulants in populations at high risk for chronic wounds [18, 19].

In this ex vivo observational study, we demonstrate that whole blood clot formation using the ActiGraft system remains feasible in patients receiving oral anticoagulation therapy, despite a measurable prolongation in clotting time compared with age-matched controls. Although anticoagulated samples exhibited a statistically significant delay in clot formation, all samples consistently formed complete, stable clots within the predefined observation window of 8–12 min. This study, nonetheless, was designed to assess the feasibility of generating autologous whole blood clots under anticoagulated conditions rather than to evaluate clinical efficacy or wound-healing outcomes.

Although significant, the observed difference in clotting time between anticoagulated participants and controls was modest, approximately 2.5 min. From a practical standpoint, this degree of prolongation is unlikely to meaningfully affect routine hospital or outpatient wound care workflows, which typically involve multi-step processes such as assessment, preparation, treatment, and documentation that extend beyond this timeframe. ActiGraft is prepared at the bedside and requires an ex vivo coagulation period of approximately 8 min according to the Instructions for Use, which allows for variability in clotting time. In this context, the observed delay should be regarded as a manageable procedural consideration rather than a functional limitation of the therapy. These findings indicate that ActiGraft preparation remains feasible in patients receiving oral anticoagulant therapy, although clinicians should be aware of the modest prolongation when planning procedural timing.

The consistent formation of a cohesive clot under anticoagulated conditions is biologically plausible. Anticoagulant therapies inhibit thrombin generation and modify fibrin clot structure, but they typically slow and weaken, rather than abolish, fibrin formation, especially under conditions of strong tissue factor-driven activation or exogenous procoagulant supplementation [20–22]. As a result, clot architecture and stability can still be achieved to produce an autologous matrix suitable for therapeutic application. In the present study, all clots generated under anticoagulated conditions were structurally intact and suitable for application as wound dressings, as measured by the clot formation time.

No significant differences in clotting time were observed between patients receiving warfarin and those receiving rivaroxaban, suggesting that the impact on whole blood clot formation is comparable across these anticoagulant classes. This finding aligns with prior evidence from other clinical contexts indicating that autologous clot- or fibrin-based biomaterials can be generated and applied safely in anticoagulated patients, although such data have largely been derived from procedural or surgical settings rather than chronic wound care [17, 23]. While clot formation was consistently achieved, variability in clotting times across groups was observed, and the relatively small sample size limits the precision of these estimates.

### Study limitations

Several limitations should be acknowledged. Firstly, the study was conducted as an exploratory ex vivo study using blood from individuals without active chronic wounds and therefore does not account for potential interactions among anticoagulation, the wound microenvironment, comorbidities, and in situ clot integration. Secondly, the modest sample size was underpowered to detect subgroup effects on clotting dynamics by anticoagulant dose, level, and clinical indication. Thirdly, the median age of the study population was 47 [42–52] years, which may limit generalizability to older patients. Older patients represent a larger proportion of individuals with chronic wounds and are more likely to receive anticoagulant therapy. Age-related changes in coagulation and wound healing may influence clot formation dynamics and should be considered in future studies. Lastly, participants were not assessed for anemia or thrombocytopenia.

## Conclusion

In summary, the prolonged clot formation time caused by oral anticoagulation with warfarin or rivaroxaban was not clinically significant as it did not impede the formation of complete, usable autologous whole blood clots with the ActiGraft system within the predefined observation window. Therefore, anticoagulation status should not preclude patients from receiving ActiGraft. Future, adequately powered studies are necessary to evaluate the ActiGraft system in real-world clinical populations. Specifically, studies should include patients with chronic wounds, a range of common comorbidities, and those receiving diverse classes of anticoagulant therapy. Moreover, larger studies should aim to assess not only clot formation dynamics but also clinical endpoints in order to clarify the broader clinical utility of ActiGraft in populations at high risk for impaired wound healing.

## Electronic Supplementary Material

Below is the link to the electronic supplementary material.

Supplementary Material 1

## Data Availability

All data supporting the findings of this study are available within the paper and its Supplementary Information.

## References

[1] Järbrink K, Ni G, Sönnergren H, Schmidtchen A, Pang C, Bajpai R, Car J (2017) The humanistic and economic burden of chronic wounds: a protocol for a systematic review. Syst Rev 6(1):15. 10.1186/S13643-016-0400-8(PubMed) PMC5259833 28118847

[2] Martinengo L, Olsson M, Bajpai R, Soljak M, Upton Z, Schmidtchen A, Car J, Järbrink K (2019) Prevalence of chronic wounds in the general population: systematic review and meta-analysis of observational studies. Ann Epidemiol 29:8–15. 10.1016/J.ANNEPIDEM.2018.10.005(PubMed) 30497932

[3] Malone M, Bjarnsholt T, McBain AJ, James GA, Stoodley P, Leaper D, Tachi M, Schultz G, Swanson T, Wolcott RD (2017) The prevalence of biofilms in chronic wounds: a systematic review and meta-analysis of published data. J Wound Care 26(1):20–25. 10.12968/jowc.2017.26.1.20(The Ohio State University) 28103163

[4] Wicke C, Bachinger A, Coerper S, Beckert S, Witte MB, Königsrainer A (2009) Aging influences wound healing in patients with chronic lower extremity wounds treated in a specialized wound care center. Wound Repair Regen 17(1):25–33. 10.1111/J.1524-475X.2008.00438.X 19152648

[5] Gould L, Abadir P, Brem H, Carter M, Conner-Kerr T et al (2015) Chronic wound repair and healing in older adults: current status and future research. Wound Repair Regen 23(1):1–13. 10.1111/WRR.12245 PMC4414710 25486905

[6] Serena TE, Kushnir I, Kushnir A, Yaakov RA, Eckert KA (2019) The safety of an autologous whole blood clot product applied to full thickness dermal wounds in a porcine model for up to 18 days. Chronic Wound Care Manage Res. 10.2147/CWCMR.S189836

[7] Schultz GS, Wysocki A (2009) Interactions between extracellular matrix and growth factors in wound healing. Wound Repair Regen 17(2):153–162. 10.1111/J.1524-475X.2009.00466.X 19320882

[8] Snyder RJ, Schultz G, Wachuku C, Rashid AM, Ead JKK (2020) Proposed mechanism of action of topically applied autologous blood clot tissue: a quintessential cellular and tissue based therapy. J Am Podiatr Med Assoc. 10.7547/20-140 33052392

[9] Snyder R, Nouvong A, Ulloa J, Bowersox K, Lopez R, Sharma A et al (2024) Efficacy and safety of autologous whole blood clot in diabetic foot ulcers: a randomised controlled trial. J Wound Care 33(9):688–700. 10.12968/JOWC.2024.0195 39287414

[10] Landau Z, Whitacre KL, Leewood C, Hawkins J, Wachuku CD (2022) Utilization of a topical autologous blood clot for treatment of pressure ulcers. Int Wound J. 10.1111/IWJ.13927 PMC9927894 36054532

[11] Heisler S, Samaan R, Lessing R, Ozker E, Snyder R (2025) Real-world outcomes of autologous whole blood clot therapy for venous leg ulcers. J Wound Care 34(10):874–880. 10.12968/JOWC.2025.0419 41071298

[12] Burnett AE, Mahan CE, Vazquez SR, Oertel LB, Garcia DA, Ansell J (2016) Guidance for the practical management of the direct oral anticoagulants (DOACs) in VTE treatment. J Thromb Thrombolysis 41(1):206–232. 10.1007/S11239-015-1310-7 PMC4715848 26780747

[13] Risser F, Urosev I, López-Morales J, Sun Y, Nash MA (2022) Engineered molecular therapeutics targeting fibrin and the coagulation system: a biophysical perspective. Biophys Rev 14(2):427–461. 10.1007/S12551-022-00950-W PMC8984085 35399372

[14] Park S, Park JK (2024) Back to basics: the coagulation pathway. Blood Res 59(1). 10.1007/S44313-024-00040-8 PMC11519239 39466528

[15] De Caterina R, Husted S, Wallentin L et al (2013) Vitamin K antagonists in heart disease: current status and perspectives (Section III). Position paper of the ESC Working Group on Thrombosis–Task Force on Anticoagulants in Heart Disease. Thromb Haemost 110(6):1087–1107. 10.1160/TH13-06-0443 24226379

[16] Perzborn E, Roehrig S, Straub A, Kubitza D, Mueck W, Laux V (2010) Rivaroxaban: a new oral factor Xa inhibitor. Arterioscler Thromb Vasc Biol 30(3):376–381. 10.1161/ATVBAHA.110.202978 20139357

[17] Katz MS, Ooms M, Heitzer M, Naderi N, Boffano P, Lauritano D et al (2024) Platelet-rich fibrin as a hemostatic agent in dental extractions in patients taking anticoagulants or antiplatelet medication: a systematic review. Clin Oral Investig 28(11):587–600. 10.1007/S00784-024-05983-X PMC11467017 39387914

[18] Alwafi H, Wei L, Naser AY, Sultan AA, Al-Hamad B et al (2020) Trends in oral anticoagulant prescribing in individuals with type 2 diabetes mellitus: a population-based study in the UK. BMJ Open 10(5):e034573. 10.1136/BMJOPEN-2019-034573 PMC7232627 32414823

[19] Badescu MC, Popescu D, Gosav EM et al (2025) The use of direct oral anticoagulants (DOACs) in the geriatric population—how to overcome the challenges of geriatric syndromes. J Clin Med 14(13):4396. 10.3390/JCM14134396 PMC12250010 40648768

[20] Dirienzo L, Vitulli A, Mancazzo F, Sacco R et al (2021) Differential effect of direct oral anticoagulants on thrombin generation and fibrinolysis in patients with atrial fibrillation and venous thromboembolism. Blood Transfus 20(6):505–512. 10.2450/2021.0153-21 PMC9726627 34967721

[21] Ataullakhanov FI, Dashkevich NM, Ovsepyan RA et al (2026) Heparin and direct oral anticoagulants have different effects on the phases of activation and spatial spread of blood coagulation. Thromb Haemost 126(1). 10.1055/A-2516-7384 PMC12758956 39961345

[22] Gauer JS, Riva N, Page EM, Barnes GD et al (2020) Effect of anticoagulants on fibrin clot structure: a comparison between vitamin K antagonists and factor Xa inhibitors. Res Pract Thromb Haemost 4(8):1269–1281. 10.1002/rth2.12443 PMC7695561 33313466

[23] de Mourão AB, Miron CF, de Mello Machado RJ, Ghanaati RC, Alves S, Calasans-Maia GG (2019) Usefulness of platelet-rich fibrin as a hemostatic agent after dental extractions in patients receiving anticoagulant therapy with factor Xa inhibitors: a case series. Oral Maxillofac Surg 23(3):381–386. 10.1007/S10006-019-00769-Y 31102080

