# Supplementary material for: Autologous whole blood clot formation with actigraft in patients receiving oral anticoagulant therapy: an ex vivo feasibility study
Source: J Thromb Thrombolysis. 2026 May 24;59(7):1960–6. doi: 10.1007/s11239-026-03321-4 (PMC13654871; doi:10.1007/s11239-026-03321-4)
Supplement: Supplementary file 1 [file 11239_2026_3321_MOESM1_ESM.docx]

Supplementary Table 1: Time to clot formation per patient (average of 2 measurements)

| Group | Clotting time (minutes) |
| --- | --- |
| Vitamin K antagonist  (Warfarin) (n=9) | 11 |
|  | 10.5 |
|  | 16.25 |
|  | 11 |
|  | 8 |
|  | 11 |
|  | 12.5 |
|  | 10.5 |
|  | 11 |
| Factor Xa inhibitor (Rivaroxaban) (n=6) | 12.75 |
|  | 13 |
|  | 10 |
|  | 17 |
|  | 12 |
|  | 11 |
| Control (n=10) | 9 |
|  | 10 |
|  | 6 |
|  | 8 |
|  | 7 |
|  | 8.5 |
|  | 8.5 |
|  | 9 |
|  | 8 |
|  | 9.5 |
